# Role of Endoplasmic Reticulum Stress-Associated Genes in Septic Neonatal Foals

**DOI:** 10.3390/antiox14081024

**Published:** 2025-08-21

**Authors:** Dipak Kumar Sahoo, David Wong, Biswaranjan Paital, Rebecca E. Ruby, Ashish Patel

**Affiliations:** 1 Department of Veterinary Clinical Sciences, College of Veterinary Medicine, Iowa State University, Ames, IA 50011, USA; 2 Redox Regulation Laboratory, Department of Zoology, College of Basic Science and Humanities, Odisha University of Agriculture and Technology, Bhubaneswar 751003, Odisha, India; biswaranjanpaital@gmail.com; 3 Veterinary Diagnostic Laboratory, Department of Veterinary Science, University of Kentucky, Lexington, KY 40511, USA; rebecca.ruby@uky.edu; 4 Department of Life Sciences, Hemchandracharya North Gujarat University, Patan 384265, Gujarat, India; uni.ashish@gmail.com

**Keywords:** sepsis, endoplasmic reticulum stress, horse, signaling pathways, oxidative stress, inflammation

## Abstract

The progression of inflammation during sepsis represents a multifaceted biological cascade that requires effective therapeutic interventions to improve survival. In septic neonatal foals, oxidative stress (OS) arises due to a compromised antioxidant defense system. Oxidative stress may disrupt the functionality of redox-sensitive organelles, such as the endoplasmic reticulum (ER). Endoplasmic reticulum stress disorder affects multiple cellular signaling pathways, including redox balance, inflammation, and apoptosis, and contributes to the pathogenesis of sepsis. The study aimed to elucidate whether OS conditions in sepsis influenced gene expression associated with ER stress. Blood samples were collected from 7 healthy and 21 hospitalized neonatal foals and processed for RNA extraction. RNA sequencing was employed to identify ER stress-responsive genes. Novel findings reported here indicate activation of the ER stress pathway in foals with sepsis. Several genes associated with ER stress, such as clusterin (*CLU*), BCL2-like 1 (*BCL2L1*), ubiquitin specific peptidase 14 (*USP14*), bifunctional apoptosis regulator (*BFAR*), and optic atrophy 1 (*OPA1*), were upregulated and positively correlated with sepsis scores and negatively correlated with the combined activities of antioxidant enzymes. In contrast, X-box binding protein 1 (*XBP1*), homocysteine inducible ER protein with ubiquitin-like domain 1 (*HERPUD1*), leucine-rich repeat kinase 2 (*LRRK2*), and selenoprotein S (*SELENOS*) were negatively correlated with sepsis scores and were downregulated in sepsis and positively correlated with the combined activities of antioxidant enzymes. Furthermore, a positive correlation was observed between cAMP responsive element binding protein 3 like 2 (*CREB3L2*) and *BCL2L1*, as well as between the expressions of *USP14* and YOD1 deubiquitinase (*YOD1*) in sepsis. Similarly, the expression levels of *XBP1* and *Herpud1* demonstrated a positive correlation with each other in sepsis. Additionally, the downregulation of genes with protective function against OS, such as *XBP1*, *HERPUD1*, and *SELENOS*, in septic foals also highlights their significance in mitigating OS in sepsis treatment. The study reported here highlights the potential of ER stress as a promising therapeutic target and prognostic marker in septic foals.

## 1. Introduction

Sepsis, defined as “life-threatening organ dysfunction caused by a dysregulated host response to infection” [1], ranks among the leading causes of mortality (in people) globally [2]. Septic shock and multiple organ failure represent the primary contributors to mortality in sepsis. In horses, despite significant progress in medical management, sepsis remains a major cause of morbidity and mortality [3,4] and is frequently encountered in neonatal foals [5,6,7]. The systemic inflammatory response to microbial invasion can result in rapid deterioration even with pathogen management, posing a significant challenge for equine practitioners [4]. The body’s inflammatory and oxidative responses in sepsis are crucial in managing infection, bacterial clearance, and promoting healing processes [8,9]. However, vigorous host responses to infection can lead to dysregulated inflammatory and oxidative responses, resulting in tissue damage, organ dysfunction, and mortality.

The body strives to regulate inflammatory and oxidative damage by preserving endogenous anti-inflammatory and antioxidant systems [8,10,11,12]. The anti-inflammatory response is often surpassed in sepsis, resulting in systemic inflammatory response syndrome (SIRS). Concurrently, when antioxidant defense levels are compromised, a redox imbalance favoring oxidative pathways and an increase in oxidative stress (OS) can occur [9,11,13]. Reactive oxygen species (ROS) and reactive nitrogen species (RNS), including superoxide, hydroxyl radicals, as well as nitric oxide and peroxynitrite, along with oxidants such as hydrogen peroxide, play significant roles in the pathogenesis of sepsis [14]. Their formation is attributed to the innate immune system, particularly the actions of neutrophils and macrophages, which contribute to the oxidative burst during the initial phases of the sepsis process [15].

While ROS are essential for routine cellular functions and can be generated in the cytosol and by various organelles (e.g., endoplasmic reticulum [ER] and mitochondria), OS can also potentially interfere with the functionality of these redox-sensitive organelles [11,12,16,17,18,19,20,21,22,23]. The ER serves as a specialized organelle responsible for folding and trafficking of proteins and exhibits a high sensitivity to alterations in intracellular homeostasis and extracellular stimuli. ER stress disorder is marked by the accumulation of misfolded proteins in the ER lumen, which significantly impacts various cellular signaling pathways, such as redox balance, inflammation, and apoptosis [24]. Cells have evolved a highly conserved signaling pathway known as the unfolded protein response (UPR), which is triggered by ER stress to address imbalanced ER protein folding, ultimately aiming to restore ER homeostasis [24]. The generation of ROS has been associated with ER stress and the UPR. Research indicates that changes in ER redox homeostasis can lead to ER stress, potentially triggering the generation of ROS in the ER and mitochondria [25]. Previous studies indicate that there are impaired antioxidant defense mechanisms, causing OS in ill and bacteremic neonatal foals [7]. In the present study, we hypothesize that the involvement of the ER stress pathway is activated in septic foals, and several genes are linked to ER stress. Here, we report multiple genes linked to ER stress exhibited upregulation, demonstrating a positive correlation with sepsis scores and a negative correlation with the overall activities of antioxidant enzymes. In contrast, the downregulation of ER stress-responsive genes, which have protective functions against OS in septic foals, underscores their importance in alleviating OS in sepsis treatment.

## 2. Materials and Methods

### 2.1. Study Design and Sample Acquisition

For the present study, a subgroup of twenty-eight foals (seven foals from each group based on sepsis score as described below) from the previous research conducted by Wong et al. (2025) was used with approval from Iowa State University (ISU) Institutional Animal Care and Use Committee (IACUC) (#22-228) [7]. Foals were classified as non-septic hospitalized controls if they had a normal physical examination, vital signs within reference intervals, reached specific benchmarks (e.g., standing, ambulating, nursing) within 2 h, had a serum IgG concentration > 800 mg/dL measured at ≤24 h of age, and had an updated sepsis score of ≤5 [7,26]. All hospitalized foals underwent blood collection for culture, as previously outlined [7]. Hospitalized foals were divided into illness groups such as hospitalized controls (sepsis score 0–5), mild (sepsis score 6–11), moderate (sepsis score 12–17), or severe (sepsis score 18–29) illness according to their sepsis scores and based on either positive or negative blood culture results [7]. Immediately following the blood draw, 2.5 mL of blood was transferred into the PAXgene Blood RNA Tube (BD Science, San Jose, CA, USA) containing 6.9 mL of RNA stabilization additive. This RNA stabilization additive ensures the immediate stabilization of intracellular RNA to yield accurate and reproducible gene expression data. The contents were mixed gently by inverting the tube 5–10 times and then positioned vertically on a wire rack or horizontally within a plastic bag for freezing. The tubes were initially frozen at −20 °C for 24 h before being transported to −80 °C in accordance with the manufacturer’s instructions (https://www.qiagen.com/us/resources/faq/3491; accessed on 15 January 2024).

### 2.2. Total RNA Extraction and Sequencing

At the end of the sample collection period, the frozen blood samples were processed for RNA extraction (Novogene Corporation Inc., Sacramento, CA, USA). Total RNA extraction was conducted using the TRIzol reagent (Invitrogen, Carlsbad, CA, USA). Though in horses, the level of globin mRNA is low [27], Zymo-Seq RiboFree Total RNA Library Kit (Zymo Research, Irvine, CA, USA) was used to remove rRNA and globin mRNA. Quality assessment and Illumina sequencing were subsequently conducted (Novogene Corporation Inc., Beijing, China). The Qubit RNA Assay Kit was employed alongside a Qubit 4.0 Fluorometer to ascertain RNA concentration, while RNA integrity was assessed using the RNA Nano 6000 Assay Kit with the Agilent Bioanalyzer 2100 system (Agilent Biotechnologies, Santa Clara, CA, USA). Sequencing libraries were created using the NEBNext Ultra II RNA Library Prep Kit for Illumina (New England Biolabs, Ipswich, MA, USA) in accordance with the manufacturer’s guidelines. The quality of the library was assessed by Qubit 4.0 Fluorometer (Life Technologies, Carlsbad, CA, USA) and real-time PCR for exact quantification, in conjunction with the Agilent Bioanalyzer 2100 system for analyzing size distribution, utilizing the Agilent RNA 6000 Nano Kit (Agilent, Santa Clara, CA, USA). Libraries that were quantified were merged and sequenced on an Illumina Novaseq6000 platform, resulting in 150 bp paired-end reads. The preliminary handling of raw data (raw reads) in FASTQ format was performed utilizing in-house Perl scripts developed by Novogene (https://www.bioz.com/result/in%E2%80%91house%20perl%20script/product/Novogene; accessed on 15 January 2024).

### 2.3. Transcriptional Analysis of Genes Involved in ER Stress

The reference genome and gene model annotation files for *Equus caballus* were sourced directly from the genome website [28]. A reference genome index was created, and paired-end clean reads were aligned to the *Equus caballus* reference genome using Hisat2 v2.0.5 [29]. Hisat2 was chosen as the mapping tool because of its ability to create a database of splice junctions using the gene model annotation file, leading to more efficient mapping results compared to other non-splice mapping tools. FeatureCounts v1.5.0-p3 was utilized to quantify the number of reads aligned to each gene [30]. In RNA-seq, the conversion to FPKM (expected number of Fragments Per Kilobase of transcript sequence per Million base pairs sequenced) [31] was conducted for the read count.

### 2.4. Statistical Analysis of the Enrichment of Differentially Expressed Genes (DEGs)

The analysis of genes that are expressed differentially was performed for various groups (control, mild, moderate, and severe) according to their sepsis scores using the DESeq2R package (1.20.0), with comparisons made between Severe vs. Control, Moderate vs. Severe, Mild vs. Severe, Moderate vs. Control, Mild vs. Control, and Mild vs. Moderate [32,33]. The *p*-values acquired were adjusted using Benjamini and Hochberg’s approach [34]. Genes with adjusted *p* ≤ 0.05, as identified by DESeq2, were categorized as differentially expressed genes (DEGs), and the criteria for significant differential expression were set at log2(fold_change) > 1 [35]. Analysis of DEGs through Gene Ontology (GO) enrichment was performed utilizing the clusterProfiler R package. GO terms with adjusted *p*-values ≤ 0.05 were considered significantly enriched by DEGs. The clusterProfiler R package was used to assess the statistical enrichment of DEGs in Kyoto Encyclopedia of Genes and Genomes (KEGG) pathways [36], while the KOBAS 2.0 web server facilitated the annotation and identification of enriched pathways and diseases [37,38]. Pearson’s correlation coefficients were used to assess the relationship between the expression levels of different ER stress-associated genes, culture status, sepsis score, and mortality, with a threshold for minimal statistical significance set at *p*  <  0.05.

### 2.5. Assessment of Oxidative Stress Markers, Activities of Antioxidant Defense Enzymes, and Histological Alterations

Malondialdehyde (MDA), recognized as a product of lipid peroxidation (LPx), was estimated in plasma samples as previously described [7] in accordance with the manufacturer’s instructions (Abcam, Waltham, MA, USA). Reactive oxygen species (ROS) such as hydrogen peroxide (H_2_O_2_) levels and the activities of antioxidant defense enzymes such as superoxide dismutase (SOD), catalase (CAT), glutathione peroxidase (GPx), and glutathione reductase (GR) in serum samples from these animals reported earlier [7] were utilized to calculate the ratios of SOD/CAT, SOD/GPx+CAT, GR/GPx and SOD+CAT+GPx+GR and correlation analysis. Pearson correlation coefficients among the expression levels of various ER stress-associated genes, culture status, sepsis score, mortality rate, oxidative stress, and antioxidant defense status were determined by GraphPad Prism 10 (GraphPad, Boston, MA, USA). A *p* < 0.05 was considered significant. A representative foal, described within the Severe illness group, was observed to be weak and considered to have a grave prognosis. Consequently, it was euthanized, and histological alterations were subsequently studied.

## 3. Results

### 3.1. Gene Ontology (GO) Functional Annotation of DEGs

DEGs were analyzed in different groups based on sepsis scores (control, mild, moderate, and severe). In the comparison between the Severe and Control groups, among 3044 DEGs, 13 were identified as ER stress genes. In the analysis of the Mild vs. Severe groups, from 2941 DEGs, 6 were ER stress genes. The Moderate vs. Severe group revealed 3 ER stress genes out of 1638 DEGs, while the Moderate vs. Control comparison showed 5 ER stress genes among 1374 DEGs. In the Mild vs. Control group, there were 1490 DEGs with 4 ER stress genes, and the Mild vs. Moderate comparison included 795 DEGs with only one ER stress gene identified (Supplemental Table S1). Since more ER stress DEGs were identified in the Severe vs. Control group, we concentrated on this group for further analysis. The DEGs (upregulated/UR and downregulated/DR) were systematically categorized based on their GO annotations. In the Severe vs. Control groups, there was a notable enrichment of significantly modulated DEGs in the biological process of (i) negative regulation of ER stress-induced intrinsic apoptotic signaling pathway (4 UR and 4 DR) and (ii) negative regulation of response to ER stress (9 UR and 4 DR) (Supplemental Table S1).

**Figure 1:**
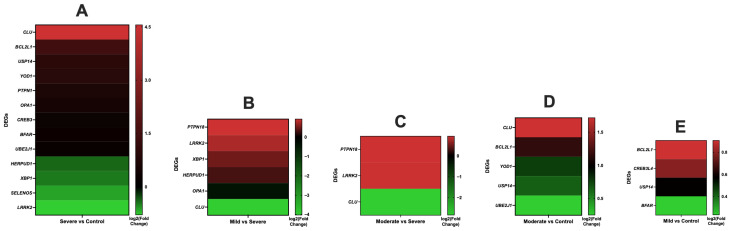
Analysis of heat maps for DEGs associated with ER stress in different treatment groups: (**A**) Severe vs. Control, (**B**) Mild vs. Severe, (**C**) Moderate vs. Severe, (**D**) Moderate vs. Control, (**E**) Mild vs. Control. Heat maps illustrate color-coded expression levels (log2-fold change) of the most significantly up- or downregulated differentially expressed genes (DEGs) in foal blood cells. More information is available in Supplemental Table S2. *Equus caballus* clusterin (*CLU*); BCL2 like 1 (*BCL2L1*); Ubiquitin specific peptidase 14 (*USP14*); X-box binding protein 1 (*XBP1*); Homocysteine inducible ER protein with ubiquitin-like domain 1 (*HERPUD1*); Leucine-rich repeat kinase 2 (*LRRK2*); Bifunctional apoptosis regulator (*BFAR*); Optic atrophy 1 (*OPA1*); cAMP responsive element binding protein 3 like 4 (*CREB3L4*); YOD1 deubiquitinase (*YOD1*); Ubiquitin-conjugating enzyme E2 J1 (*UBE2J1*); Mitochondrial dynamin-like GTPase, Protein tyrosine phosphatase, non-receptor type 1 (*PTPN1*); cAMP responsive element binding protein 3 (*CREB3*); Selenoprotein S (*SELENOS*); Endoplasmic Reticulum (ER).

**Figure 2:**
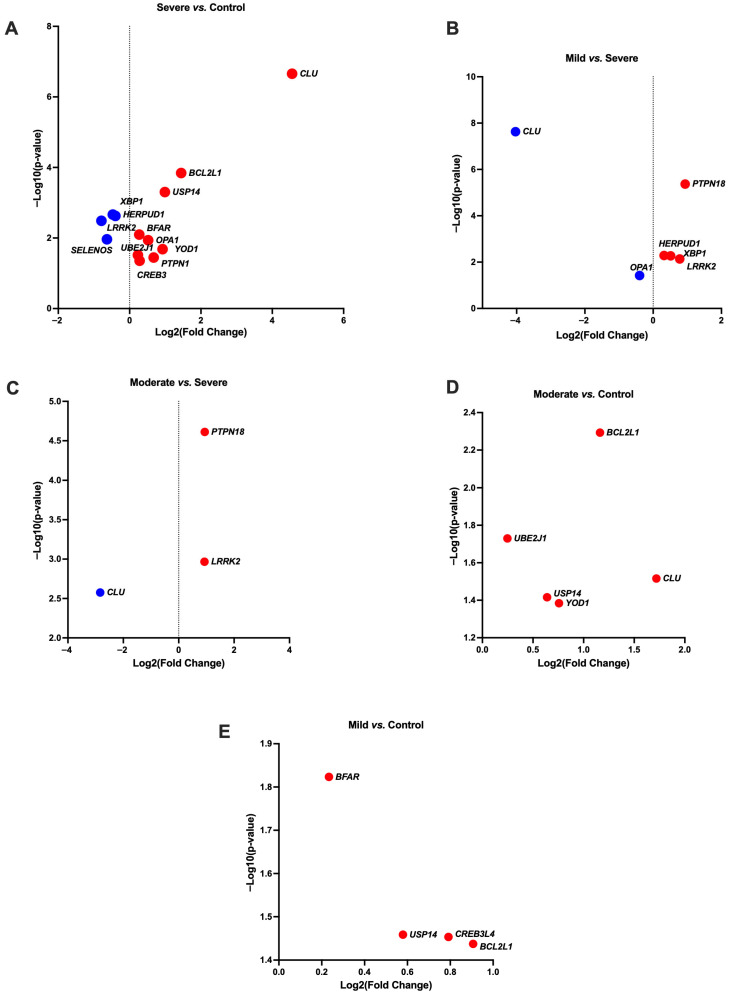
Volcano plots showing gene expression differences between different treatment groups (**A**) Severe vs. Control, (**B**) Mild vs. Severe, (**C**) Moderate vs. Severe, (**D**) Moderate vs. Control, (**E**) Mild vs. Control. Upregulated DEGs are represented by red dots, and downregulated DEGs are represented by blue dots. More information is available in Supplemental Table S2. *Equus caballus* clusterin (*CLU*); BCL2 like 1 (*BCL2L1*); Ubiquitin specific peptidase 14 (*USP14*); X-box binding protein 1 (*XBP1*); Homocysteine inducible ER protein with ubiquitin-like domain 1 (*HERPUD1*); Leucine-rich repeat kinase 2 (*LRRK2*); Bifunctional apoptosis regulator (*BFAR*); Optic atrophy 1 (*OPA1*); cAMP responsive element binding protein 3 like 4 (*CREB3L4*); YOD1 deubiquitinase (*YOD1*); Ubiquitin-conjugating enzyme E2 J1 (*UBE2J1*); Mitochondrial dynamin-like GTPase, Protein tyrosine phosphatase, non-receptor type 1 (*PTPN1*); cAMP responsive element binding protein 3 (*CREB3*); Selenoprotein S (*SELENOS*); Endoplasmic reticulum (ER).

The genes associated with ER stress encompassed *Equus caballus* clusterin (*CLU*), BCL2-like 1 (*BCL2L1*), ubiquitin specific peptidase 14 (*USP14*), X-box binding protein 1 (*XBP1*), homocysteine inducible ER protein with ubiquitin-like domain 1 (*HERPUD1*), leucine-rich repeat kinase 2 (*LRRK2*), bifunctional apoptosis regulator (*BFAR*), optic atrophy 1 (*OPA1*), cAMP responsive element binding protein 3 like 4 (*CREB3L4*), syntaxin binding protein 1 (*STXBP1*), YOD1 deubiquitinase (*YOD1*), ubiquitin-conjugating enzyme E2 J1 (*UBE2J1*), mitochondrial dynamin-like GTPase, protein tyrosine phosphatase, non-receptor type 1 (*PTPN1*), cAMP responsive element binding protein 3 (*CREB3*), and selenoprotein S (*SELENOS*) (Table 1, Figure 1 and Figure 2 and Supplemental Table S2).

In the Severe vs. Control group, nine genes exhibited increased expression, including *CLU*, *BCL2L1*, *USP14*, *YOD1*, *PTPN1*, *OPA1*, *CREB3*, *BFAR*, and *UBE2J1*, whereas four genes, namely *HERPUD1*, *XBP1*, *SELENOS* and *LRRK2*, showed decreased expression (Table 1, Figure 1 and Figure 2 and Supplemental Table S2). Notably, in the Mild vs. Severe group, the expression of five genes, including *PTPN18*, *LRRK2*, *XBP1*, and *HERPUD1*, was increased, whereas two genes, namely *OPA1* and *CLU*, were downregulated (Table 1, Figure 1 and Figure 2 and Supplemental Table S2). Markedly, in the Mild vs. Moderate group, *STXBP1* was observed to be downregulated. Conversely, four genes, *BCL2L1*, *CREB3L4*, *USP14*, and *BFAR*, showed upregulation in the Mild vs. Control group. In the comparison between the Moderate and Control groups, five genes exhibited upregulation, including *CLU*, *BCL2L1*, *YOD1*, *USP14*, and *UBE2J1*. In the Moderate vs. Severe group, two genes displayed increased expression, namely *PTPN18* and *LRRK2*, whereas *CLU* showed decreased expression (Table 1, Figure 1 and Figure 2 and Supplemental Table S2).

### 3.2. Gene Expression Correlation Analysis

**Figure 3:**
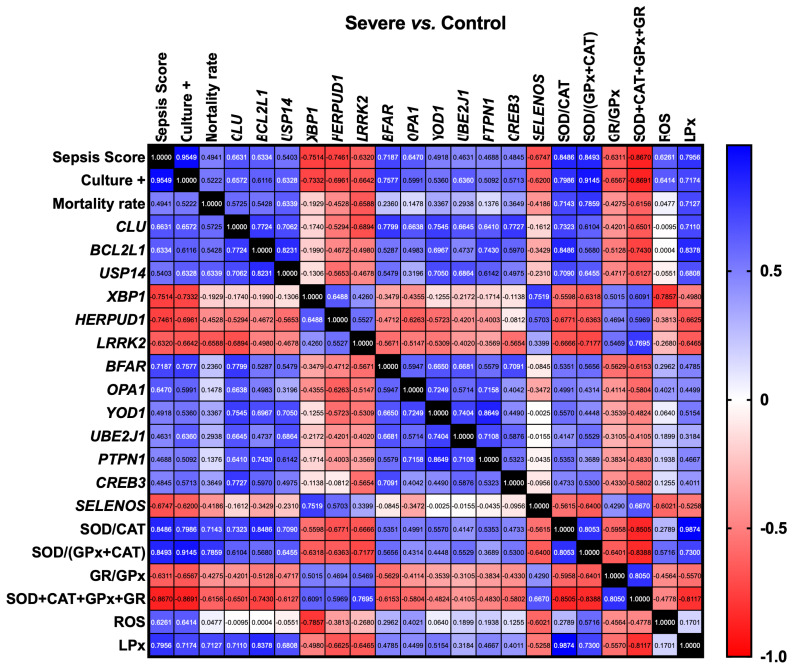
A heat map illustrating the Pearson correlation coefficients among the expression levels of various ER stress-associated genes, culture status, sepsis score, mortality rate, oxidative stress, and antioxidant defense status in Severe vs. Control groups. A threshold for minimal statistical significance was established at *p* < 0.05. Positive correlations are represented in blue, while negative correlations are indicated in red. The value 1 signifies a “perfect” positive correlation and is represented in black. More information is available in Supplemental Table S3, including the details on *p*-values. *Equus caballus* clusterin (*CLU*); BCL2 like 1(*BCL2L1*); Ubiquitin specific peptidase 14 (*USP14*); X-box binding protein 1 (*XBP1*); Homocysteine inducible ER protein with ubiquitin-like domain 1 (*HERPUD1*); Leucine-rich repeat kinase 2 (*LRRK2*); Bifunctional apoptosis regulator (*BFAR*); Optic atrophy 1 (*OPA1*); YOD1 deubiquitinase (*YOD1*); Ubiquitin-conjugating enzyme E2 J1 (*UBE2J1*); Mitochondrial dynamin-like GTPase, Protein tyrosine phosphatase, non-receptor type 1 (*PTPN1*); cAMP responsive element binding protein 3 (*CREB3*); Selenoprotein S (*SELENOS*); Endoplasmic Reticulum (ER); Superoxide dismutase (SOD); Catalase (CAT); Glutathione peroxidase (GPx); Glutathione reductase (GR); Reactive oxygen species (ROS); Lipid peroxidation (LPx).

In the Severe vs. Control group, the genes associated with ER stress, including *CLU* (*p* = 0.01), *BCL2L1* (*p* = 0.015), *USP14* (*p* = 0.046), *BFAR* (*p* = 0.004), and *OPA1* (*p* = 0.012), demonstrated a significant positive correlation with sepsis score. Also, a significant positive correlation was observed for these genes with the SOD/CAT ratio (*CLU*, *p* = 0.003; *BCL2L1*, *p* = 0.0001; *USP14*, *p* = 0.005; *BFAR*, *p* = 0.048), as well as with the SOD/CAT+GPx ratio (*CLU*, *p* = 0.02; *BCL2L1*, *p* = 0.034; *USP14*, *p* = 0.013; *BFAR*, *p* = 0.035) and the OS indicator LPx (*CLU*, *p* = 0.004; *BCL2L1*, *p* = 0.0002; *USP14*, *p* = 0.007). Conversely, a significant negative correlation was noted with the SOD+CAT+GPX+GR combination (*CLU*, *p* = 0.01; *BCL2L1*, *p* = 0.002; *USP14*, *p* = 0.02) (Figure 3).

**Figure 4:**
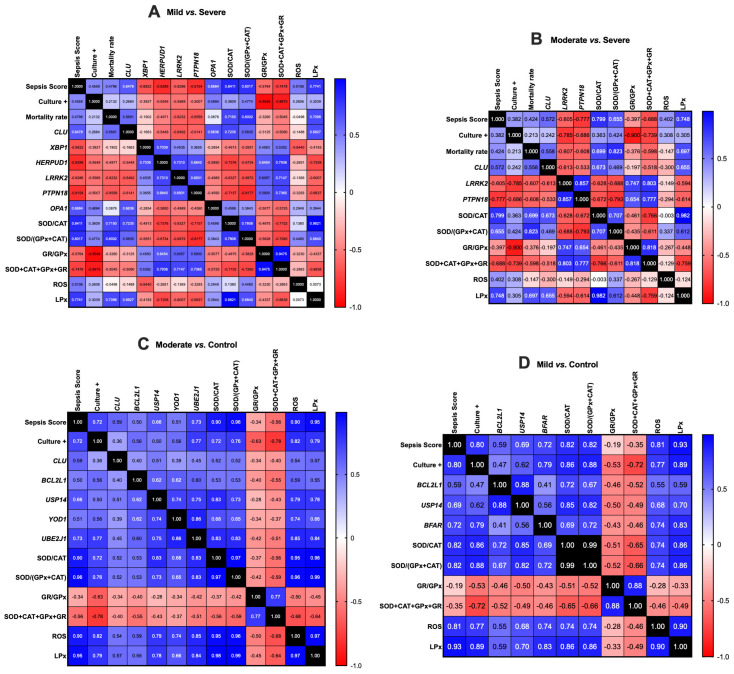
A heat map illustrating the Pearson correlation coefficients among the expression levels of various ER stress-associated genes, culture status, sepsis score, mortality rate, oxidative stress, and antioxidant defense status in (**A**) Mild vs. Severe, (**B**) Moderate vs. Severe, (**C**) Moderate vs. Control, and (**D**) Mild vs. Control groups. More information is available in Supplemental Table S4A–D, including the details on *p*-values. *Equus caballus* clusterin (*CLU*); BCL2 like 1 (*BCL2L1*); Ubiquitin specific peptidase 14 (*USP14*); X-box binding protein 1 (*XBP1*); Homocysteine inducible ER protein with ubiquitin-like domain 1 (*HERPUD1*); Leucine-rich repeat kinase 2 (*LRRK2*); Bifunctional apoptosis regulator (*BFAR*); Optic atrophy 1 (*OPA1*); YOD1 deubiquitinase (*YOD1*); Ubiquitin-conjugating enzyme E2 J1 (*UBE2J1*); Mitochondrial dynamin-like GTPase, Protein tyrosine phosphatase, non-receptor type 1 (*PTPN1*); Endoplasmic Reticulum (ER); Superoxide dismutase (SOD); Catalase (CAT); Glutathione peroxidase (GPx); Glutathione reductase (GR); Reactive oxygen species (ROS); Lipid peroxidation (LPx). Positive correlations are represented in blue, while negative correlations are indicated in red. The value 0.99 or 1 signifies a “perfect” positive correlation and is represented in black.

In contrast, *XBP1* (*p* = 0.002), *HERPUD1* (*p* = 0.002), *LRRK2* (*p* = 0.015), and *SELENOS* (*p* = 0.008) showed a significant negative correlation with sepsis score. ER stress-associated genes, including *CLU* (*p* = 0.011), *BCL2L1* (*p* = 0.02), *USP14* (*p* = 0.015), *BFAR* (*p* = 0.002), *OPA1* (*p* = 0.024), *YOD1* (*p* = 0.048), *UBE2J1* (*p* = 0.014), and *CREB3* (*p* = 0.018), demonstrated a significant positive correlation with culture positivity. In contrast, *XBP1* (*p* = 0.003), *HERPUD1* (*p* = 0.006), *LRRK2* (*p* = 0.01), and *SELENOS* (*p* = 0.018) showed a significant negative correlation with culture-positive score (Figure 3 and Figure 4 and Supplemental Tables S3 and S4). Also, a significant negative correlation was noted for these genes with the SOD/CAT ratio (*XBP1*, *p* = 0.037; *HERPUD1*, *p* = 0.008; *LRRK2*, *p* = 0.009; *SELENOS*, *p* = 0.037), as well as with SOD/CAT+GPx (*XBP1*, *p* = 0.015; *HERPUD1*, *p* = 0.014; *LRRK2*, *p* = 0.004; *SELENOS*, *p* = 0.014), the OS marker LPx (*HERPUD1*, *p* = 0.01; *LRRK2*, *p* = 0.012) and ROS levels (*XBP1*, *p* = 0.0009; *SELENOS*, *p* = 0.023). On the other hand, a significant positive correlation was observed with SOD+CAT+GPX+GR (*HERPUD1*, *p* = 0.024; *LRRK2*, *p* = 0.0013) (Figure 3).

Notably, *CLU* (*p* ≤ 0.013), *BCL2L1* (*p* ≤ 0.006), *USP14* (*p* ≤ 0.019), *YOD1* (*p* ≤ 0.006), and *PTPN1* (*p* ≤ 0.019) exhibited a significant positive correlation with one another. Similarly, a significant positive correlation was observed among *CLU* (*p* ≤ 0.01), *BFAR* (*p* ≤ 0.043), *USP14* (*p* ≤ 0.043), and *UBEJ1* (*p* ≤ 0.01). A significant positive correlation was also observed between *OPA1* and *CLU* (*p* ≤ 0.01). *CREB3* (*p* ≤ 0.024), *CLU* (*p* = 0.001) and *BCL2L1* (*p* ≤ 0.024) exhibited positive correlations. *XBP1* and *HERPUD1* (*p* = 0.012) exhibited a negative correlation with sepsis score while demonstrating a significant positive correlation with one another. Similarly, *HERPUD1* (*p* ≤ 0.035) and *USP14* (*p* ≤ 0.035) exhibited a positive correlation with one another (Figure 3 and Figure 4 and Supplemental Tables S3 and S4). A significant positive correlation was marked between *CLU* and SOD/CAT, as well as SOD/CAT+GPx, in the Mild vs. Severe group (Figure 4A). Conversely, *HERPUD1*, *LRRK2*, and *PTPN18* exhibited a negative correlation with SOD/CAT, SOD/CAT+GPx, and LPx, while demonstrating a positive correlation with GR/GPX and SOD+CAT+GPx+GR (Figure 4A). In a similar manner, in the Moderate vs. Severe group, *LRRK2* and *PTPN18* exhibited a negative correlation with SOD/CAT, SOD/CAT+GPx, and LPx. However, a significant positive correlation was observed between *CLU* and LPx (Figure 4B). In the Moderate vs. Control group, a significant positive correlation was observed for *CLU*, *BCL2L1*, *USP14*, *YOD1*, and *UBE2J1* with ROS and LPx. Additionally, *USP14*, *YOD1*, and *UBE2J1* exhibited a significant positive correlation with SOD/CAT and SOD/CAT+GPx (Figure 4C). Similarly, in the Mild vs. Control group, it was shown that *BCL2L1*, *USP14*, and *BFAR* exhibited a significant positive correlation with ROS, LPx, and the SOD/CAT and SOD/CAT+GPx ratios (Figure 4D).

**Figure 5:**
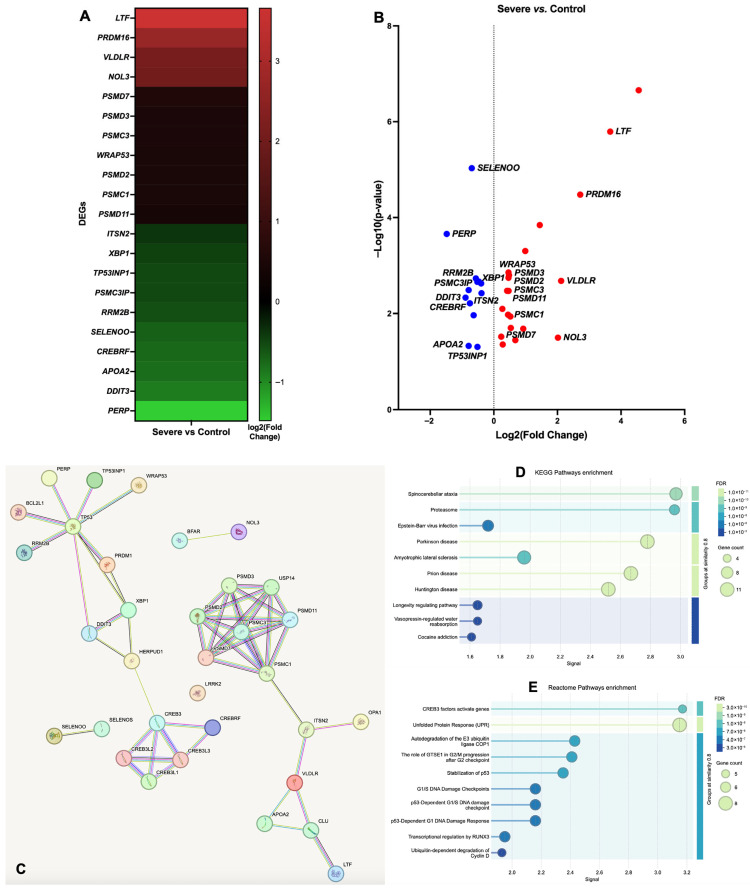

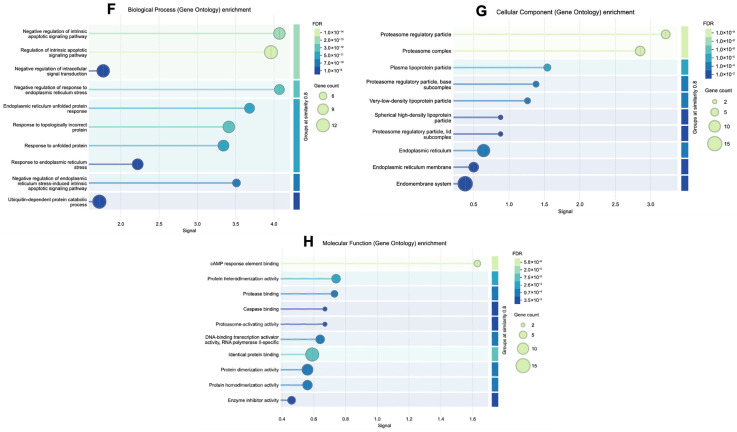
Analysis of genes associated with ER stress-responsive genes in Severe vs. Control groups. (**A**) Heat map analysis. Heat maps show color-coded expression levels (log2-fold change) of the most significantly up- or downregulated DEGs in foal blood cells. More information is available in Supplemental Table S5. (**B**) Volcano plots showing gene expression differences between the treatment groups. (**C**) Gene association network constructed using bioinformatics data mining tool STRING (https://string-db.org/; accessed on 14 June 2025) [39]. (**D**) KEGG pathway enrichment analysis. (**E**) Reactome pathway enrichment analysis. (**F**,**H**) Gene Ontology (GO) analysis. The DEGs are annotated according to (**F**) biological process, (**G**) cellular component, and (**H**) molecular function categories. Lactotransferrin (*LTF*); PR/SET domain 16 (*PRDM16*); very-low-density lipoprotein receptor (*VLDLR*); nucleolar protein 3 (*NOL3*); proteasome 26S subunit, non-ATPase 7 (*PSMD7*); proteasome 26S subunit, non-ATPase 3 (*PSMD3*); proteasome 26S subunit, ATPase 3 (*PSMC3*); WD repeat containing antisense to TP53 (*WRAP53*); proteasome 26S subunit, non-ATPase 2 (*PSMD2*); proteasome 26S subunit, ATPase 1 (*PSMC1*); proteasome 26S subunit, non-ATPase 11 (*PSMD11*); intersectin 2 (*ITSN2*); X-box binding protein 1 (*XBP1*); tumor protein p53 inducible nuclear protein 1 (*TP53INP1*); PSMC3 interacting protein (*PSMC3IP*); ribonucleotide reductase regulatory TP53 inducible subunit M2B (*RRM2B*); selenoprotein O (*SELENOO*); CREB3 regulatory factor (*CREBRF*); apolipoprotein A2 (*APOA2*); DNA damage inducible transcript 3 (*DDIT3*); p53 apoptosis effector related to PMP22 (*PERP*).

This study also identified the relationships among various ER stress genes and their interactions with several other genes, as demonstrated using STRING database (https://string-db.org/; accessed on 14 June 2025) [39,40]. ER stress-response genes interacted with various genes, such as PR/SET domain 16 (*PRDM16*), WD repeat containing antisense to TP53 (*WRAP53*), lactotransferrin (*LTF*), and very-low-density lipoprotein receptor (*VLDLR*), which showed elevated expression levels in septic foals. On the other hand, genes like intersectin 2 (*ITSN2*), PSMC3 interacting protein (*PSMC3IP*), selenoprotein O (*SELENOO*), p53 apoptosis effector related to PMP22 (*PERP*), ribonucleotide reductase regulatory TP53 inducible subunit M2B (*RRM2B*), and CREB3 regulatory factor (*CREBRF*) exhibited reduced expression in septic foals (Figure 5 and Supplemental Table S5). Additionally, the analysis included the enrichment of DEGs in the Kyoto Encyclopedia of Genes and Genomes (KEGG) pathways [36,41]. The KEGG pathway enrichment analysis offers a comprehensive organization and assessment of the results, categorizing related genes within the same pathway [36]. The KEGG analysis comparing the Severe and Control groups for ER stress genes and their associated genes revealed enrichment in pathways related to spinocerebellar ataxia (SCA), proteasome, Epstein–Barr virus (EBV) infection, Parkinson’s disease (PD), amyotrophic lateral sclerosis (ALS), prion disease, Huntington disease (HD), longevity regulating pathway, vasopressin-regulated water reabsorption, and cocaine addiction (Figure 5).

### 3.3. Histological Changes

**Figure 6:**
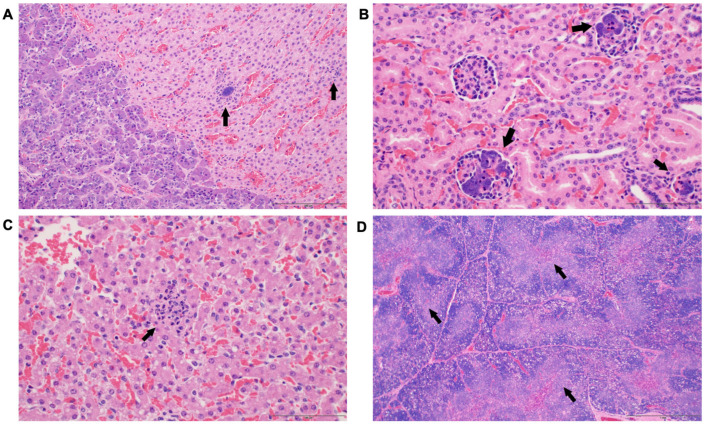
Histological alterations observed in septic foals. (**A**) Representative images of hematoxylin and eosin (H&E) staining sections of the adrenal gland under 20× magnification. Small foci of necrosis and neutrophilic inflammation are present within the adrenal cortex surrounding a small aggregate of bacteria (*Actinobacillus* sp.) (arrow). The scale bar represents 200 μm. (**B**) Representative images of H&E staining sections of the kidney at 40× magnification. Multiple capillaries within renal glomeruli contain bacterial (*Actinobacillus* sp.) colonies (arrows). Low numbers of neutrophils are infrequently observed in the adjacent glomerular regions. The scale bar represents 100 μm. (**C**) Representative images displaying H&E staining sections of liver under 40× magnification. Small, random foci of necrosis and neutrophilic inflammation are present within the hepatic parenchyma (arrow). Rarely, bacteria (*Actinobacillus* sp.) were observed within these foci. The scale bar represents 100 μm. (**D**) Representative images showing H&E staining sections of the thymus at 4× magnification. There is a diffuse, moderate decrease in lymphocytes within the thymic medulla and cortex (arrows). The scale bar represents 1 mm.

Among the 28 foals, 7 served as healthy controls, while 20 out of 21 foals (95.24%) exhibited positive bacterial growth in blood culture (Supplemental Table S6). Histological alterations in septic foals were also studied. Necropsy was performed on a representative ill and bacteremic foal, which revealed minimal macroscopic findings with microscopic findings of glomerulonephritis, adrenocortical necrosis with suppurative inflammation, and hepatitis. All affected organs had intralesional bacteria with morphology consistent with *Actinobacillus* (Figure 6). *Actinobacillus* sp. was cultured from the liver, lung and umbilicus.

## 4. Discussion

The development of inflammation in sepsis represents a multifaceted biological cascade that, in certain circumstances, can contribute to the demise of the host. Intense inflammation, marked by a cytokine storm, OS, and neutrophil accumulation, plays a crucial role in the organ failure associated with sepsis [8]. In our previous study, we observed that increased levels of several OS markers, including MDA, protein carbonyl, and H_2_O_2_ levels, combined with decreased activities of key antioxidant defense enzymes such as SOD, catalase, GR, GPx, and lower levels of reduced glutathione, suggested oxidative damage in bacteremic and ill foals [7]. The purpose of the project described here was to elucidate whether OS conditions in sepsis influenced gene expression associated with ER stress.

The responses to ER stress initiate with (1) a decrease in translation to limit the influx of nascent protein, (2) the upregulation of ER chaperones to assist in the translocation of the folded protein to the Golgi complex, (3) an increase in ER-associated protein degradation (ERAD) to eliminate unfolded or misfolded proteins; and if these issues persist, (4) the activation of apoptosis [42]. The responses are governed by the protein sensors localized in the ER. When the ER stress surpasses the compensatory abilities of UPR or becomes prolonged, apoptosis is initiated by activating cellular injuries, potentially leading to cell death. Consequently, it has been observed that ER stress and UPR are associated with various pathological and inflammatory conditions [43]. ER stress may serve as either a contributing factor or a consequence of sepsis. While the harmful impact of ER stress during infections has been established, there is increasing evidence that ER stress plays a role in sepsis pathogenesis. Specific pathological conditions, including sepsis, trauma, ischemia, and viral infection, result in the accumulation of unfolded or misfolded proteins, disrupting the homeostasis of the ER and inducing ER stress [44,45]. Several studies on ER stress signaling have uncovered an intriguing interaction between ER stress and cell death associated with sepsis [42,43,46]. Previous studies have also indicated that alleviating ER stress can enhance protein conformation stability, aid in the transport of mutated proteins, and boost ER folding capacity and have proposed that targeting ER stress response may offer therapeutic benefits for a range of conditions, including sepsis [42,47,48]. For example, in the CLP murine model of sepsis, it has been observed that ER stress plays a role in abnormal lymphocyte apoptosis during sepsis. This indicates that the apoptosis pathway mediated by ER stress could serve as a potential target for clinical prevention and treatment of sepsis-induced lymphocyte apoptosis [43].

We employed RNA sequencing (RNA-seq) to identify ER stress-responsive genes, as it serves as a method for quantifying gene expression and is frequently applied to detect differentially expressed genes (DEGs) [49,50]. The RNA-seq methods and the associated data analysis techniques are sufficiently reliable that they do not consistently necessitate validation through quantitative PCR (qPCR) or alternative methods. A number of studies specifically addressed the correlation between findings from RNA-seq and qPCR. For example, a comprehensive analysis was conducted by Everaert et al. [51], comparing five RNA-seq analysis pipelines with wet-lab qPCR results for over 18000 protein-coding genes. Although this research utilizes RNA samples from human origin [51], there is no indication that the results would vary for studies involving other organisms.

Sepsis in neonatal foals can present in various forms [4]; however, the foals involved in the present study exhibited bacteraemia [7]. The interplay between ER stress, OS, and the inflammatory response plays a vital role in the pathogenesis of a variety of diseases [50]. The equilibrium among the primary antioxidant enzymes, such as SOD, GPx, GR and CAT, is thought to be more significant than the function of individual enzymes. For instance, the ratio of SOD/CAT or even SOD/CAT+GPx has demonstrated superior antioxidant capability compared to either individual enzyme [52]. Also, GR/GPx ratio was reported to be higher in healthy subjects, and it exhibits a negative correlation with the levels of MDA [53]. The current investigation revealed a positive correlation between *CLU*, *BCL2L1* and *USP14* with the SOD/CAT and SOD/CAT+GPx ratios, as well as with LPx. On the other hand, a negative correlation for these enzymes was observed with the combined activities of SOD, CAT, GPX, and GR. While CLU serves as a highly sensitive biosensor for elevated OS levels, its regulation by OS serves as a fundamental connection across various pathological conditions associated with *CLU*. CLU promotes apoptosis following exposure to stress or an apoptosis-inducing agent. Elevated levels of intracellular CLU can become cytotoxic when they accumulate in the intracellular compartment [54,55,56]. A contrasting effect of *Bcl-2* that elevates cellular levels of ROS has been documented in HL-60 cells lacking glutathione, as well as in bacteria, astrocytes, various cancer cell lines, and fibroblasts. Bcl-2 may induce a slight rise in cellular OS, which in turn enhances the antioxidant capacity of the cell, thereby improving its resilience to further OS [57]. The inhibition of USP14 led to faster degradation of oxidized proteins and increased resistance against OS [58].

The levels of *CLU*, *BCL2L1*, *USP14*, *PTPN1*, *OPA1*, and *CREB3* were elevated during sepsis and demonstrated a positive correlation with sepsis score. CLU is a multifunctional protein that mediates cellular responses linked to organ failure, systemic inflammation, and metabolic changes. The plasma concentration of CLU was significantly higher in critically ill patients compared to healthy individuals [59], which aligns with the current study that demonstrates increased CLU expression, albeit at the transcriptional level, in foals suffering from sepsis. In addition, the present study demonstrated that both *CREB3L2* and *BCL2L1* were upregulated and were identified as positively correlated with one another as well as with the sepsis score. *CREB3L2* shares homology with *CREB3*, which is regulated by MAPK signaling [60], while *BCL2L1* exhibits a 44% homology to its important paralog *BCL-2* [61]. CREB3 is a member of the ATF6 family and functions as a transmembrane protein in the ER. Following exposure to Golgi stress inducers, CREB3 undergoes translocation from the ER to the Golgi apparatus, where it is cleaved by S1P and S2P proteases, subsequently activating the transcription of ARF4 and leading to Golgi stress-induced apoptosis. Both endogenous full-length CREB3 and CREB3L2 are targets for ER-associated protein degradation [62]. Studies have shown that the activation of RAS/MAPK or PI3K signaling cascade results in the induction of the transcription factor CREB3L2, which in turn directly activates the expression of activating transcription factor 5 (ATF5). ATF5, known for its significant role in enhancing cell survival, subsequently facilitates this process by upregulating myeloid cell leukemia sequence 1 (MCL1), a member of the anti-apoptotic B-cell leukemia/lymphoma 2 (BCL2) family [63]. Reports indicate that *CREB3* is activated in response to ER stress [64], with the Golgi membrane and S1P and S2P proteases being absorbed by the ER membrane following BFA treatment. This process leads to the cleavage of ATF6 family proteins [65], including CREB3 [66]. Bifunctional apoptosis regulator (BFAR) modulates apoptosis, cross-linking both cytosolic and mitochondrial apoptosis pathways [67]. The structure includes a DED-like domain that can inhibit apoptosis signaling via Fas (cytosolic), along with another domain that facilitates interactions with Bcl-2 family members and prevents Bax-induced apoptosis (mitochondrial) [68]. Conditions linked to BCL2L1 encompass absolute glaucoma and B-cell lymphoma. Among its associated pathways are nuclear events mediated by NFE2L2 and death receptor signaling pathways. During sepsis, proteins associated with cell death are pivotal in host immune signaling [69]. BCL-2 serves as an important regulator of cellular longevity and is influenced by interactions between pathogens and hosts, as well as by specific cytokines that are activated following viral infection [70]. A few trials involving BCL-2 inhibitors have been conducted, demonstrating their effectiveness in promoting apoptosis and improving disease outcomes [70].

In this study, both *USP14* and *YOD1* exhibited increased levels of expression, and their expression showed a positive correlation. Research indicates that YOD1 and USP21 participate in a reciprocal deubiquitination process; YOD1 influences the protein stability of USP21, whereas USP21 does not have a regulatory impact on YOD1. The combined activities of YOD1 and USP21 synergistically affect cell proliferation [71]. USP14, belonging to the USP family, is known for its role in catalyzing the cleavage of ubiquitin from various protein substrates. In the current study, *USP14* expression was also increased in septic foals. Research has shown that targeting *USP14* may serve as a potential strategy for treating sepsis [72]. The activation of the NF-κB pathway occurred through the inhibitory effect of USP14 on inhibitor of nuclear factor kappa B (I-κB) expression [73]. Neochromine S5 (S5) significantly reduced M1-like macrophage polarization, leading to a decrease in pro-inflammatory cytokines and a downregulation of nuclear factor kappa-light-chain-enhancer of activated B-cells (NF-κB) and signal transducer and activator of transcription 1 (STAT1) signaling pathways. The inhibition of USP14 enhances autophagy in M1-like macrophages and mitigates CLP-induced sepsis. Targeting USP14 by S5 to enhance autophagy presents a promising therapeutic approach for sepsis [72].

Research findings demonstrate a significant increase in YOD1 expression during sepsis, which correlates with macroautophagy. Deubiquitinase YOD1 is involved in the process of ERAD [74] and has been recognized as a potential diagnostic and prognostic biomarker for sepsis, likely influencing immune processes associated with the condition. Only YOD1 demonstrated a correlation with patient survival outcomes. Gene set enrichment analysis indicated that YOD1 plays a significant role in severe infection, macroautophagy, and immune-related mechanisms. *YOD1* also exhibited increased levels in the murine model of sepsis, consistent with the findings of the current study, and was demonstrated to effectively diagnose sepsis and predict the prognosis [75]. Moreover, YOD1 provides a defense against methicillin-resistant *Staphylococcus aureus* (MRSA) sepsis. NLR-family pyrin domain-containing-3 (*NLRP3*) inflammasome is important for the host’s defense mechanisms against microbial pathogens, and it plays a significant role in endotoxin-induced coagulation by enhancing tissue factor expression, partially via the activation of IL-1β release [76]. YOD1 primarily inhibits coagulation by suppressing the activation of the NLRP3 inflammasome. The administration of MCC950, a highly potent and specific inhibitor of NLRP3, effectively blocked YOD1-mediated coagulation in MRSA-induced sepsis, thereby supporting the role of YOD1 in regulating NLRP3 inflammasome-dependent coagulation during MRSA-induced disseminated intravascular coagulation [77].

Similarly to the rise in PTPN1 observed in septic foals in the study presented here, murine studies have demonstrated a similar pattern of a progressive increase in PTP1B levels in rat brains following the induction of sepsis. The dysfunction of brain mitochondria in the LPS-induced sepsis murine model is partially attributed to a reduction in the tyrosine phosphorylation of mitochondrial proteins, which is influenced by tyrosine kinase Src and PTP1B [78]. Deleting the *PTP1B* gene offers protection against cardiovascular dysfunction and mortality induced by septic shock, likely due to a significant decrease in cardiovascular inflammation and improvement in endothelial function [79]. *PTP1B*(−/−) mice exhibited diminished LPS-induced cardiac expression of tumor necrosis factor-α (TNF-α) and interleukin1-β. PTP1B deficiency also led to a decrease in cardiac P38 levels. Moreover, *PTP1B*(−/−) mice exhibited a significantly lower mortality rate induced by LPS, a phenomenon similarly noted with a pharmacological PTP1B inhibitor [79]. The mitochondrial dynamics in alveolar macrophages are linked to acute lung injury (ALI) induced by sepsis. Dynamin-like GTPase, optic atrophy protein 1 (OPA1), is directly associated with and undergoes deacetylation by sirtuin (SIRT)3 in alveolar macrophages. In alveolar macrophages of sepsis-induced ALI, there was a noted decrease in SIRT3 protein expression alongside an increase in OPA1 acetylation. Imbalanced mitochondrial dynamics stimulate pro-inflammatory polarization of alveolar macrophages in sepsis-induced ALI, while the deacetylation of optic atrophy protein 1 (OPA1) facilitated by SIRT3 enhances MD equilibrium, thus alleviating lung injury [80]. UBE2J1 is a ubiquitin E2 protein that plays an important role in the ER quality control system for proteasomal degradation [81], and its involvement in RNA virus infection has been reported, suggesting it may facilitate RNA virus replication [82]. The findings demonstrated that silencing *UBE2J1* significantly reduced DENV infection, whereas *UBE2J1* overexpression increased DENV infection. The expression of type I IFN was elevated in *UBE2J1*-silenced cells, while it was decreased in cells with *UBE2J1* overexpression. UBE2J1 facilitates the ubiquitination and subsequent degradation of transcription factor IFN regulatory factor 3 (IRF3), which results in the negative regulation of type one IFN expression, consequently enhancing RNA virus infection [82].

In the present study, several genes associated with ER stress, including *XBP1*, *HERPUD1*, *LRRK2*, and *SELENOS*, were downregulated in foals suffering from sepsis, showing a negative correlation with the sepsis score. Additionally, *XBP1*, *HERPUD1*, *LRRK2*, and *SELENOS* were negatively correlated with the SOD/CAT and SOD/CAT+GPx ratios, while *XBP1* and *SELENOS* were negatively correlated with ROS levels. However, *HERPUD1* and *LRRK2* exhibited a negative correlation with LPx, while showing a positive correlation with the combined activities of SOD, CAT, GPX, and GR. Numerous research findings suggest that a lower SOD/CAT ratio signifies diminished OS [83]. A decrease in OS levels is influenced by reduced SOD/CAT or SOD/GPX+CAT ratios, elevated GR/GPX ratios, or enhanced combined activities of antioxidant defense enzymes [52,53]. The overexpression of *XBP1*, which restores CAT expression in *XBP1*-deficient cells and reduces ROS generation after H_2_O_2_ exposure, along with the analysis of mutations in the catalase promoter region, suggests that XBP1 plays a protective role against OS. Its positive regulation of catalase expression potentially may be a contributing factor to this protective function [84]. Similarly, HERPUD1 plays cytoprotective roles against ER stress by reducing the activity of various caspases, preventing the collapse of mitochondrial potential, and decreasing the activation of c-Jun N-terminal kinase (JNK) [85]. HERPUD1-mediated cytoprotective effect in response to OS depends on the inositol 1,4,5-trisphosphate receptor (ITPR) and the transfer of Ca^2+^ from the ER to mitochondria. *HERPUD1* provides a protective mechanism against apoptosis induced by OS by downregulating ITPR [85]. The overexpression of mutant *LRRK2* has been linked to OS, and it has been reported that antioxidants can alleviate LRRK2 toxicity [86], while SELENOS has been observed to have inhibitory effects on inflammation and OS [87].

Selenoprotein Sl (SEPS1) belongs to the selenoprotein family, encompassing enzymes such as thioredoxin reductase and glutathione peroxidase [88]. SEPS1 has been recognized as a protein associated with ER stress response, which is likely linked to an inflammatory response [89,90]. The region of human chromosome 15, which houses *SEPS1*, has been previously proposed to harbor quantitative trait loci that affect inflammatory disorders [90]. Research indicates a significant correlation between genetic variation in the *SEPS1* gene and the circulating levels of pro-inflammatory cytokines in human populations. Furthermore, SEPS1 may play a role in controlling cytokine production in cultured macrophage cells [91], creating a regulatory loop in which cytokines enhance the expression of *SEPS1*, subsequently suppressing cytokine production. The impact of *SEPS1* knockdown using small interfering RNA (siRNA) was evaluated in a murine model of sepsis induced by lipopolysaccharide (LPS) [90], showing a reduction in *SEPS1* expression and the subsequent production of TNF α and IL 6 observed in the *SEPS1* siRNA group, which could be linked to the activation of the p38 MAPK pathway. Also, the pathological findings indicated significant lesions in liver and lung cells in the *SEPS1* siRNA group, suggesting that the *SEPS1* gene plays a protective role in the livers and lungs of mice affected by sepsis [90].

In the present septic foal study, expression levels of both *XBP1* and *Herpud1* decreased and exhibited a negative correlation with the sepsis score while showing a positive correlation with one another. The ER possesses a regulatory mechanism known as UPR, which is triggered to restore ER homeostasis and mitigate additional cellular damage [92]. The function of the p300/XBP1s/Herpud1 axis in infiltrating macrophages has been elucidated [93]. Herpud1 is an ER-resident membrane protein and is involved in the signaling pathway of ER-associated degradation and in the maintenance of ER homeostasis [93,94] and is significantly induced by ER stress in numerous pathological conditions [95,96,97]. Homocysteine-induced ER protein (HERP) is responsible for degrading unfolded and misfolded proteins through the ERAD pathway, which serves as the primary mechanism for degrading misfolded proteins, and *HERP* knockout leads to the accumulation of ERAD substrates [98]. The ER stress activator tunicamycin enhances *HERP* expression, while the ER stress inhibitor tauroursodeoxycholic acid reduces *HERP* expression. Moreover, depletion of HERP triggers the ER stress pathway and apoptosis, while overexpression of HERP suppresses the ER stress pathway and apoptosis [97]. Nrf1 transcription factor induces the expression of Herpud1 when encountered with ER stress. The histone acetyltransferase p300 increases the stability of spliced X-box binding protein 1 (XBP1s) and boosts the transcriptional activity of the XBP1s target gene *Herpud1* [93]. *Herpud1* is transcribed by the transcription factor XBP1, which has been demonstrated to bind with high affinity to *Herpud1* promoter. XBP1 serves as an effector central to the UPR [93] and is activated by the ER stress sensor inositol-requiring enzyme 1α (IRE1α), a transmembrane protein kinase located in the ER that functions as a bifunctional enzyme. IRE1α enzyme oligomerizes in response to the accumulation of unfolded proteins in the ER lumen and cleaves the UPR-specific transcription factor, *XBP1* mRNA, leading to the formation of its active spliced variant, XBP1s [99]. XBP1s can elevate the expression of chaperones, consequently improving the protein folding capacity of the ER [99]. The unspliced XBP1 (XBP1u) and histone deacetylase 3 (HDAC3) play a crucial role in mitigating OS caused by disturbed flow through the upregulation of mammalian target of rapamycin complex 2 (mTORC2)-dependent Akt1 [100]. The presence of XBP1 was important for enhancing histone deacetylase 3 (HDAC3) protein levels. The overexpression of *XBP1u* and/or HDAC3 led to the activation of Akt1 phosphorylation, stabilization, and nuclear translocation of NF-E2-related factor 2 (Nrf2) protein, as well as the expression of heme oxygenase 1 (HO-1) [100]. XBP1 influences various aspects of disease [101]. The expression of UPR genes was associated with the progression of organ failure and endothelial dysfunction in patients experiencing sepsis [102,103]. The UPR may be implicated in these phenomena, as indicated in the sepsis model, where lymphocytes exhibited increased levels of apoptosis and expression of UPR genes [43].

LRRK2 is a large multidomain protein that encompasses, among other domains, a kinase and a GTPase domain [104] and serves as a pivotal regulator of vesicular trafficking, infection, immunity, and inflammation. Mutations in the *LRRK2* gene were identified as a potential risk factor for inflammatory bowel disease (IBD) and Crohn’s disease. For example, the *N2081D LRRK2* mutation, linked to increased Crohn’s disease risk, is located in the kinase domain and is thought to enhance kinase activity [105]. Numerous investigations have highlighted the role of *LRRK2* in relation to infection, especially in response to bacterial pathogens [106]. For instance, an in vitro study indicates that LRRK2 plays a role in innate immunity, demonstrating its contribution to the restriction of the enteric pathogen *Salmonella* by macrophages. The expression of *LRRK2* increases NF-κB-dependent transcription, indicating its involvement in immune response signaling. The endogenous LRRK2 protein rapidly translocates near bacterial membranes, and the knockdown of *LRRK2* disrupts ROS production during the processes of phagocytosis and bacterial elimination [107]. Mice deficient in *LRRK2* exhibit increased vulnerability to peritoneal inflammation, leading to compromised control of *Salmonella* and elevated mortality rates in infected mice. Also, inhibition of LRRK2 kinase by GSK2578215A enhances the susceptibility of mice to *Salmonella* infection [108]. Also, LRRK2 influences the release of the anti-inflammatory cytokine IL-10 in mouse macrophages following infection with *Mycobacterium tuberculosis*. During chronic *M. tuberculosis* infection in mice, the lack of *LRRK2* leads to increased secretion of pro-inflammatory cytokines, characterized by elevated levels of IFN-γ [109].

Using STRING database, this research further elucidated the relationships between different ER stress genes and their interactions with several other genes. STRING (https://string-db.org/, accessed on 14 June 2025) systematically compiles and integrates interactions between proteins, encompassing both physical interactions and functional relationships, specifically gathering gene expression data from RNA expression arrays and RNA-Seq datasets curated by GEO database as well as co-regulation data sourced from the ProteomeHD database [40]. Moreover, STRING is notable due to its diverse sources of evidence, its rigorous scoring system, user-friendly interface, and comprehensive suite of enrichment features [39]. In the present research, several genes linked to ER stress-response genes were identified, including *PRDM16*, *WRAP53*, *LTF*, and *VLDLR*, which exhibited increased expression in sepsis. Conversely, genes such as *ITSN2*, *PSMC3IP*, *SELENOO*, *PERP*, *RRM2B*, and *CREBRF* showed decreased expression in septic foals. The *CREBRF* expression was found to be reduced, whereas CREB3 levels were increased in septic foals. ER stress-associated transcription factor CREB3 plays a crucial role in maintaining the homeostasis of Ca^2+^, ATP, and ROS, as knockout of *Creb3* leads to increased vulnerability to H_2_O_2_ and elevated basal levels of ROS. The rise in CREB3 may also result from a reduction in CREBRF levels. CREBRF functions as a negative regulator of CREB3 by directing nuclear CREB3 to discrete foci within the nucleus, resulting in the degradation of CREB3 protein and the suppression of CREB3-mediated activation of promoters that include the unfolded protein response element (UPRE) [110]. RRM2B serves as the DNA damage-inducible small subunit of ribonucleotide reductase, which is the rate-limiting enzyme in the synthesis of de novo deoxyribonucleoside triphosphates [111]. RRM2B inhibits the activation of the OS pathway, and the decreased levels of RRM2B observed in the present study may contribute to increased OS. The observed reduction in *PERP* in this study may be attributed to a cell-type-specific role for Perp in the p53 cell death pathway. Perp plays a selective role in mediating the p53 apoptotic response, with its necessity determined by the specific cellular context [112]. The reduction in *SELENOO* activity observed in septic foals may be attributed to lower serum selenium levels. The selenium acquired through dietary sources is subsequently employed to synthesize selenoproteins, which play a crucial role in carrying out the biological functions associated with selenium [113]. The metabolism of selenium is disturbed during sepsis, leading to a reduction in serum selenium levels caused by the diminished synthesis of Selenoprotein P [SelP], the selenoprotein that plays a crucial role in selenium transport. The expression of other selenoproteins depends on the availability of selenium for Sec biosynthesis within tissue-specific locations [113]. The expressions of *PSMC3IP* and *ITSN2* were also found to be reduced in septic foals. *PSMC3* proteasome subunit variants are linked to the production of type I interferon, which can worsen bacterial infections [114]. Long non-coding RNA intersectin 1-2 (lnc-ITSN1-2) is associated with inflammation, multiple organ dysfunction, and an increased risk of mortality in patients with sepsis [115]. In septic foals, several genes, including *PRDM16*, *WRAP53*, *LTF*, and *VLDLR*, were activated to address inflammation or enhance survival rates in sepsis. The present study also identified a notable increase in *VLDLR* expression during sepsis. Lipopolysaccharide may be sequestered in adipose tissue through the very-low-density lipoprotein receptor, and this sequestration could play a role in enhancing survival rates during sepsis. LTF has demonstrated significant involvement as a key innate immune responder and has played a crucial role in regulating the progression of acute septic inflammation [116]. LTF exhibits serine protease activity, capable of cleaving arginine-rich regions found in various microbial virulence proteins. This function played a role in the regulation of antimicrobial activity [116]. Neutrophils have the capability to directly generate LTF, and the release of LTF is crucial in both the progression and resolution of inflammation [116]. PRDM16 interacts directly with the promoter regions of GPX4, enhancing its expression. PRDM16 plays a role in inhibiting ferroptosis through the NRF2/GPX4 axis or GPX4, thereby helping to prevent multi-organ injury induced by sepsis, which includes acute kidney injury (AKI) [117]. The ablation of PRDM16 from kidney proximal tubules in mice resulted in a reduction in NRF2 and GPX4 expression, which caused a lower glutathione (GSH)/oxidized glutathione (GSSG) ratio, an increase in reactive oxygen species (ROS) production, and advancement of AKI [117]. The increased expression of *WRAP53β* results in accelerated repair processes and enhanced cell survival [118].

The KEGG analysis conducted between the Severe and Control groups for ER stress genes and their associated genes indicated significant enrichment in pathways linked to SCA, proteasome, EBV infection, PD, ALS, prion disease, HD, longevity regulating pathway, vasopressin-regulated water reabsorption, and cocaine addiction. The unfolded protein response (UPR) plays an important role in determining lifespan by regulating ER stress [119]. The ubiquitin-proteasome system (UPS) plays a crucial role in the degradation of over 80% of cellular proteins; however, ER stress exerts a broad inhibitory influence on the UPS [120]. Reports indicate that the proteolytic activity of the proteasome was elevated in skeletal muscle from patients with sepsis and multiple organ failure [121]. Both ER stress and proteasome dysfunction arise during the pathogenesis of spontaneous prion diseases [122]. Protein misfolding and aggregation, which result in ER stress, are key contributors to the pathogenic mechanisms observed in neurodegenerative diseases, including PD [123], ALS due to the disruption of proteostasis [124], HD [125], and SCA [126]. The process by which neurodegeneration occurs due to cocaine abuse has been linked to neuroinflammation related to advanced HIV-1 infection [127]. Additionally, it has been noted that cocaine can induce autophagy through the activation of ER stress pathways upstream [127]. Acute-onset ataxia resulting from sepsis has been documented [128]. Patients with PD exhibit a higher susceptibility to sepsis [129], and in hospitalized ALS patients, sepsis often emerges as a common cause of mortality [130]. The relationship between ER stress and EBV lytic gene expression is well-established [131]. Reactivation of EBV is frequently observed in patients experiencing sepsis [132]. The relationship between ER stress in arginine vasopressin neurons [133] and elevated arginine vasopressin levels in cases of hemorrhagic and septic shock [134] has been documented. All these studies indicate a connection between these KEGG pathways and sepsis and ER stress genes.

## 5. Conclusions

The present results demonstrate that the ER stress pathway is activated in foals experiencing sepsis. This study presents findings on several genes linked to ER stress, including *CLU*, *BCL2L1*, *USP14*, *BFAR*, and *OPA1*, which showed a positive correlation with sepsis score and a negative correlation with the combined activities of antioxidant enzymes. Conversely, *XBP1*, *HERPUD1*, *LRRK2*, and *SELENOS* exhibited a negative correlation with sepsis scores and a positive correlation with the combined activities of antioxidant enzymes, highlighting the potential of ER stress as a novel therapeutic target and prognostic marker in septic foals. Moreover, the downregulation of genes with a protective role against OS, such as *XBP1*, *HERPUD1*, and *SELENOS* in septic foals, underscores their importance in alleviating OS in addressing sepsis. Exploring pharmacological strategies to target the ER stress pathway and improving cytoprotective function could provide valuable insights for the treatment and prevention of sepsis, especially considering this pathway’s role in disease progression. In conclusion, these findings enhance the mechanistic understanding of sepsis in neonatal foals and propose the ER stress pathway as a potential biomarker or target for future therapeutic interventions, not only in equine medicine but potentially in broader veterinary or comparative medical applications.

## Figures and Tables

**Table 1:** Details of DEGs associated with ER stress in different treatment groups.

Group Comparisons	Gene ID	Gene Name	Chromosome	Gene Start Position	Gene End Position	Gene Description	log2 (Fold Change)	*p*-Value	−log10 (*p*-Value)
Severe vs. Control	ENSECAG00000007010	*CLU*	2	56547399	56619680	*Equus caballus* clusterin, mRNA. [Source: RefSeq mRNA; Acc: NM_001081944]	4.555	2.21 × 10^−7^	6.656
	ENSECAG00000017223	*BCL2L1*	22	23334139	23376133	BCL2 like 1 [Source: VGNC Symbol; Acc: VGNC:15791]	1.445	1.44 × 10^−4^	3.843
	ENSECAG00000022985	*USP14*	8	43792907	43839139	Ubiquitin specific peptidase 14 [Source: VGNC Symbol; Acc: VGNC:24832]	0.989	4.99 × 10^−4^	3.302
	ENSECAG00000009722	*YOD1*	5	3273118	3275205	YOD1 deubiquitinase [Source: HGNC Symbol; Acc: HGNC:25035]	0.925	0.021	1.683
	ENSECAG00000019772	*PTPN1*	22	39442194	39520318	Protein tyrosine phosphatase, non-receptor type 1 [Source: VGNC Symbol; Acc: VGNC:22010]	0.675	0.036	1.445
	ENSECAG00000024248	*OPA1*	19	33025234	33104793	*OPA1*, mitochondrial dynamin like GTPase [Source: VGNC Symbol; Acc: VGNC:21035]	0.520	0.012	1.939
	ENSECAG00000000184	*CREB3*	25	999440	1002983	cAMP Responsive element binding protein 3 [Source: VGNC Symbol; Acc: VGNC:56871]	0.280	0.044	1.355
	ENSECAG00000016920	*BFAR*	13	31568145	31593542	Bifunctional apoptosis regulator [Source: VGNC Symbol; Acc: VGNC:15818]	0.271	0.008	2.097
	ENSECAG00000012492	*UBE2J1*	10	42734584	42758609	Ubiquitin conjugating enzyme E2 J1 [Source: VGNC Symbol; Acc: VGNC:24721]	0.232	0.030	1.517
	ENSECAG00000011366	*HERPUD1*	3	9952325	9962055	Homocysteine inducible ER protein with ubiquitin like domain 1 [Source: VGNC Symbol; Acc: VGNC:18755]	−0.398	0.002	2.627
	ENSECAG00000014780	*XBP1*	8	10108130	10114753	X-box binding protein 1 [Source: HGNC Symbol; Acc: HGNC:12801]	−0.471	0.002	2.664
	ENSECAG00000012386	*SELENOS*	1	107309263	107317239	Selenoprotein S [Source: VGNC Symbol; Acc: VGNC:22803]	−0.636	0.011	1.963
	ENSECAG00000010288	*LRRK2*	6	60224506	60356951	Leucine-rich repeat kinase 2 [Source: VGNC Symbol; Acc: VGNC:19801]	−0.791	0.003	2.489
Mild vs. Severe	ENSECAG00000011090	*PTPN18*	18	211051	255397	Protein tyrosine phosphatase, non-receptor type 18 [Source: VGNC Symbol; Acc: VGNC:22015]	0.938	4.27 × 10^−6^	5.369
	ENSECAG00000010288	*LRRK2*	6	60224506	60356951	Leucine-rich repeat kinase 2 [Source: VGNC Symbol; Acc: VGNC:19801]	0.780	0.007	2.136
	ENSECAG00000014780	*XBP1*	8	10108130	10114753	X-box binding protein 1 [Source: HGNC Symbol; Acc: HGNC:12801]	0.512	0.005	2.271
	ENSECAG00000011366	*HERPUD1*	3	9952325	9962055	Homocysteine inducible ER protein with ubiquitin like domain 1 [Source: VGNC Symbol; Acc: VGNC:18755]	0.320	0.005	2.287
	ENSECAG00000024248	*OPA1*	19	33025234	33104793	*OPA1*, mitochondrial dynamin like GTPase [Source: VGNC Symbol; Acc: VGNC:21035]	−0.397	0.038	1.421
	ENSECAG00000007010	*CLU*	2	56547399	56619680	*Equus caballus* clusterin (*CLU*), mRNA. [Source: RefSeq mRNA; Acc: NM_001081944]	−4.031	2.352 × 10^−8^	7.629
Moderate vs. Severe	ENSECAG00000011090	*PTPN18*	18	211051	255397	Protein tyrosine phosphatase, non-receptor type 18 [Source: VGNC Symbol; Acc: VGNC:22015]	0.939	2.444 × 10^−5^	4.612
	ENSECAG00000010288	*LRRK2*	6	60224506	60356951	Leucine-rich repeat kinase 2 [Source: VGNC Symbol; Acc: VGNC:19801]	0.930	0.001	2.967
	ENSECAG00000007010	*CLU*	2	56547399	56619680	*Equus caballus* clusterin (*CLU*), mRNA. [Source: RefSeq mRNA; Acc: NM_001081944]	−2.834	0.003	2.576
Moderate vs. Control	ENSECAG00000007010	*CLU*	2	56547399	56619680	*Equus caballus* clusterin (*CLU*), mRNA. [Source: RefSeq mRNA; Acc: NM_001081944]	1.720	0.030	1.516
	ENSECAG00000017223	*BCL2L1*	22	23334139	23376133	BCL2 like 1 [Source:VGNC Symbol;Acc:VGNC:15791]	1.162	0.005	2.293
	ENSECAG00000009722	*YOD1*	5	3273118	3275205	YOD1 deubiquitinase [Source: HGNC Symbol; Acc: HGNC:25035]	0.757	0.041	1.384
	ENSECAG00000022985	*USP14*	8	43792907	43839139	Ubiquitin specific peptidase 14 [Source: VGNC Symbol; Acc: VGNC:24832]	0.639	0.038	1.416
	ENSECAG00000012492	*UBE2J1*	10	42734584	42758609	Ubiquitin conjugating enzyme E2 J1 [Source: VGNC Symbol; Acc: VGNC:24721]	0.247	0.019	1.730
Mild vs. Moderate	ENSECAG00000013078	*STXBP1*	25	31639631	31702301	Syntaxin binding protein 1 [Source: VGNC Symbol; Acc: VGNC: 23736]	−1.412	0.042	1.374
Mild vs. Control	ENSECAG00000017223	*BCL2L1*	22	23334139	23376133	BCL2 like 1 [Source: VGNC Symbol; Acc: VGNC:15791]	0.907	0.037	1.437
	ENSECAG00000017552	*CREB3L4*	5	40245551	40249420	cAMP Responsive element binding protein 3 like 4 [Source: VGNC Symbol; Acc: VGNC:16856]	0.792	0.035	1.454
	ENSECAG00000022985	*USP14*	8	43792907	43839139	Ubiquitin specific peptidase 14 [Source:VGNC Symbol; Acc: VGNC:24832]	0.579	0.035	1.459
	ENSECAG00000016920	*BFAR*	13	31568145	31593542	Bifunctional apoptosis regulator [Source: VGNC Symbol; Acc: VGNC:15818]	0.234	0.015	1.823

Endoplasmic reticulum (ER); differentially expressed genes (DEGs).

## Data Availability

All the data are available in tables, figures, and Supplemental Information.

## Supplementary Materials

The following supporting information can be downloaded at: https://www.mdpi.com/article/10.3390/antiox14081024/s1, Supplemental Table S1. Details on Gene Ontology (GO) analysis of DEGs in septic foals. Supplemental Table S2. DEGs associated with ER stress in individual foals from different septic groups. Supplemental Table S3. Details on correlation analysis in Severe vs. Control group. Supplemental Table S4. Details on correlation analysis in (A) Mild vs. Severe, (B) Moderate vs. Severe, (C) Moderate vs. Control and (D) Mild vs. Control groups. Supplemental Table S5. DEGs associated with ER stress-responsive genes in individual foals in Severe vs. Control groups. Supplemental Table S6. Blood culture results in individual foals from different septic groups.
